# Association between Occupational Dysfunction and Metabolic Syndrome in Community-Dwelling Japanese Adults in a Cross-Sectional Study: Ibara Study

**DOI:** 10.3390/ijerph15112575

**Published:** 2018-11-17

**Authors:** Yuki Miyake, Eri Eguchi, Hiroshi Ito, Kazufumi Nakamura, Tatsuo Ito, Kenjiro Nagaoka, Noriyoshi Ogino, Keiki Ogino

**Affiliations:** 1 Department of Public Health, Okayama University Graduate School of Medicine, Dentistry and Pharmaceutical Sciences, 2-5-1 Shikata-cho, Kita-ku, Okayama 700-8558, Japan; miyake-y@kiui.ac.jp (Y.M.) eri_eguchi@okayama-u.ac.jp (E.E.); praesepe_m44@icloud.com (T.I.); nagaokak@okayama-u.ac.jp (K.N.); n-ogino@med.uoeh-u.ac.jp (N.O.); 2 Department of Occupational Therapy, School of Health Sciences and Social Welfare, Kibi International University, 8 Iga-machi, Takahashi, Okayama 716-8508, Japan; 3 Department of Cardiovascular Medicine, Okayama University Graduate School of Medicine, Dentistry and Pharmaceutical Sciences, 2-5-1 Shikata-cho, Kita-ku, Okayama 700-8558, Japan; itomd@md.okayama-u.ac.jp (H.I.); ichibun@cc.okayama-u.ac.jp (K.N.)

**Keywords:** occupational dysfunction, metabolic syndrome, community-dwelling people

## Abstract

The purpose of this study was to investigate the relationship between occupational dysfunction and metabolic syndrome (MetS) and its component factors in community-dwelling Japanese adults (N = 1,514). Self-reported lifestyle behaviors, Classification and Assessment of Occupational Dysfunction (CAOD) scores, and metabolic traits were measured. CAOD levels were divided into tertiles (low, moderate, and high), and their associations with MetS and its components were evaluated through logistic regression analysis. The association of MetS with CAOD was demonstrated in the total number of individuals [OR = 1.92 (95% CI 1.17–3.17)] and in older individuals [OR = 1.90 (95% CI 1.04–3.46)]. The association of dyslipidemia and CAOD was evident for overweight individuals [OR = 2.08 (95% CI 1.17–3.68)]. A higher association of high blood pressure with CAOD was evidenced in younger individuals [OR = 2.02 (95% CI 1.05–3.89)] who belonged to the highest-CAOD-score group in comparison to those who registered the lowest-CAOD-score group. The evaluation of MetS and interventions related to its prevention may be more effective if the viewpoint of occupational dysfunction is taken into account.

## 1. Introduction

Positive psychological factors such as ikigai, life enjoyment, life satisfaction, or happiness are associated with greater longevity, reduced risk of cardiovascular disease, and reduced risk of physical disability [1,2,3,4,5]. Negative psychological factors, such as anger, anxiety, hopelessness, or psychological stress are associated with an increased risk of cardiovascular disease and mortality [6,7,8,9].

In the field of occupational therapy, negative aspects of the human lifestyle are called occupational dysfunction [10]. Occupational dysfunction was originally proposed as a concept by Kielhofner in the Model of Human Occupation (MOHO) [11,12]. Occupational dysfunction is recognized worldwide as a major health-related problem [11,12,13]. An occupation is considered to be the center of the human experience [10]. It includes the activities that people need to, want to, and are expected to do [14]. An occupation involves not just work, business, and labor but also a wide range of conduct such as education, play, activities of daily living, rest, and social participation [10]. Occupational dysfunction is a state in which daily activities (work, leisure, self-care, and rest) are not properly performed [11]. Occupational dysfunction is defined as a negative experience related to engaging in daily activities, and has four components; occupational marginalization, occupational imbalance, occupational alienation, and occupational deprivation [10]. Occupational marginalization is defined as a person not having the opportunity to engage in desired daily activities. Occupational imbalance is defined as a loss of balance in engaging in daily activities. Occupational alienation is defined as a situation when the inner needs of the individual for daily activities are not satisfied. Occupational deprivation is defined as a lack of opportunity for daily activities beyond the individual’s control. These occur in both disabled and nondisabled people, and result in a worsening sense of well-being and deteriorating physical health [15,16]. In observational studies, the prevalence of occupational dysfunction was 36% for office workers [17], and 75.4% for healthcare workers without obvious medical disease [18].

Occupational dysfunction causes a decrease in physical activity. It is believed that decreasing physical activity tends to cause obesity, and results in MetS. Therefore, occupational dysfunction and MetS are considered to be closely associated. An association between occupational dysfunction and high blood pressure, body mass index (BMI), and lack of sleep for healthcare workers has previously been demonstrated by the authors of the present paper although the subject population scale was small and the occupation of the participants was limited to healthcare [18]. MetS is a problem that affects the entirety of society, and not just healthcare professionals. Therefore, it was considered necessary to investigate the relationship between occupational dysfunction and MetS.

In this study, we aimed to investigate the relationship between occupational dysfunction and MetS in community-dwelling Japanese men and women. We hypothesized that occupational dysfunction is associated with MetS and its components.

## 2. Methods

### 2.1. Ethics Statement

This research protocol was approved by the Ethics Committee of Okayama University Graduate School of Medicine, Dentistry, and Pharmaceutical Sciences (No. 1506-079) and the study conformed to the Declaration of Helsinki guidelines. We provided participants with a letter explaining the conduct and purpose of the study and obtained their written informed consent. 

### 2.2. Participants

A total of 2,218 adults received an annual health checkup in 2014 in Ibara city, Okayama, Japan. Of these 1,668 provided informed consent to their participation in this study, a response rate of 75.2%. 154 subjects were excluded because of missing data for age, sex, BMI, high-density lipoprotein cholesterol (HDL-C), triglyceride (TG), blood pressure (BP), hemoglobin A1c (HbA1c), or Classification and Assessment of Occupational Dysfunction (CAOD) score. A total of 1,514 participants were included in the final analysis.

### 2.3. Measurements

BP was measured twice in a sitting position after resting using an automated sphygmomanometer. The mean of the two measurements was used for analysis. Blood samples were collected after a period of overnight fasting. Serum and plasma were used to measure HDL-C, low-density lipoprotein cholesterol (LDL-C), TG, and HbA1c. 

MetS was defined using the modified Japanese criteria [19]. As we did not have information on waist circumference, we defined overweight as BMI ≥ 25 kg/m^2^, in line with a previous study [20]. In this study, MetS was defined as BMI ≥ 25 kg/m^2^ plus any two of the following factors: (1) HDL-C <40 mg/dL, triglycerides ≥150 mg/dL, and/or medication for dyslipidemia, (2) systolic blood pressure (SBP) ≥130 mmHg, diastolic blood pressure (DBP) ≥85 mmHg, and/or medication for hypertension, (3) HbA1c ≥ 6.0% (NGSP) and/or medication for glucose intolerance.

### 2.4. Lifestyle Behavior

Study participants completed a self-administered questionnaire including information on age, sex, smoking habits, regular alcohol intake, exercise habits, sleeping, education status, and marital status. These variables were dichotomized: smoking habits (current smoker or nonsmoker), regular alcohol intake (current drinker or nondrinker), exercise habits (exerciser or non-exerciser), sleeping (deficient sleep or sufficient sleep), education status (up to junior high or higher than junior high), and marital status (living with a spouse or other [single, divorced, or widowed]).

### 2.5. Classification and Assessment of Occupational Dysfunction (CAOD)

The CAOD was developed to evaluate occupational dysfunction, based on a new Occupational-Based Practice (OBP 2.0) [10,21,22]. It has been widely used to assess occupational dysfunction [18,23]. The reliability and validity of the CAOD were confirmed in studies using university students [10]. Construct validity, structural validity, hypothesis testing (convergent and discriminant validity), internal consistency reliability, concurrent validity, predictive validity, test-retest reliability, and item response were analyzed [10,24,25]. This scale also indicated transferability in healthy adults [18].

The CAOD contains 16 items across four factors with a 7-point response scale ranging from 1 (disagree) to 7 (agree). CAOD comprises four factors: occupational marginalization (6 items), occupational imbalance (4 items), occupational alienation (3 items), and occupational deprivation (3 items). The highest possible score was 112, and the lowest possible score was 16. The cutoff value was set at 52 points [24]. However, the evaluation of the cutoff point was conducted only for healthcare workers. In this study, confirmatory factor analysis (CFA) of CAOD had 16 items and 4 factors (Comparative Fit Index; CFI = 0.961, Root Mean Square Error of Approximation; RMSEA = 0.072). The result for internal consistency, ω coefficient, of the CAOD total score was 0.85, and all the factors were within an acceptable range. There are several ways to determine the possibility of occupational dysfunction [18,25].

### 2.6. Statistical Analysis

The researchers first calculated the mean age, the age, and sex-adjusted mean values, the prevalence of clinical parameters, and the lifestyles of subjects according to CAOD score categories. CAOD scores were divided into tertile as low (16–19), moderate (20–30), or high (≥ 31). Next, the linear trend was assessed through a regression model. When the values of CAOD scores were categories divided into tertiles, the differences in the mean values of several clinical parameters were analyzed by age- and sex-adjusted ANCOVA. After the ANCOVA was accomplished, multiple comparisons (Bonferroni) were performed.

Logistic regression models were used to calculate unadjusted and multivariable-adjusted odds ratios (ORs) and 95% confidence intervals (CIs) for MetS and each component factors, according to CAOD score categories. Multivariable adjustment included sex, age, BMI, smoking habits, regular alcohol intake, sleeping, exercise habits, marital status, and education status. A linear regression analysis was performed using linear trends across CAOD categories with the median score of CAOD for each category.

We then conducted the same analysis stratified by overweight status (non-overweight [BMI < 25 kg/m^2^] and overweight [BMI ≥ 25 kg/m^2^]), and age categories (younger [< 65 years old] and older [≥ 65 years old]). Then, we repeated the analysis for the non-overweight and older individuals, overweight and older individuals, non-overweight and younger individuals, and overweight and younger individuals. 

Tests for a statistical interaction between overweight status and CAOD score categories, age categories and CAOD score categories, overweight status and CAOD score categories in the older individuals, and overweight status and CAOD score categories in the younger individuals on MetS, and each component factors was conducted by entering an interaction term for overweight status or age categories and measures of CAOD (overweight status × CAOD score categories, age categories × CAOD score categories) in a multivariate model.

We used the statistical analysis software (SAS) program Ver. 9.4 (SAS Institute Inc., Cary, NC, USA) and Windows SPSS (version 22.0; SPSS Japan Inc., Tokyo, Japan) for all statistical analysis. All probability values for statistical tests were 2-tailed, and *p* values < 0.05 were regarded as statistically significant.

## 3. Results

Table 1 displays the mean age, the age, and sex-adjusted mean values, prevalence of clinical parameters, and lifestyles according to CAOD score categories. The numbers of the score categories of 16–19, 20–30, and ≥ 31 were respectively 489, 516, and 509. Compared to people belonging to the reference 16–19 score category, those who registered the highest CAOD scores were more likely to be younger, to not exercise habitually, to get deficient sleep, to have a lower BMI, and to record lower SBP, DBP, and LDL. Age- and sex-adjusted ANCOVA was performed on the values of clinical parameters in CAOD scores. BMI, DBP, and LDL-C were significantly different. With respect to lifestyle factors, exercise habits, and deficiency of sleep were significantly dissimilar.

Multiple comparisons revealed a significant disparity in BMI, DBP, LDL-C, and exercise habits, and recorded differences in the quality of sleep between the lowest-CAOD-score group and the highest-CAOD-score group.

The prevalence of occupational dysfunction in this study was 4.7%, and the mean age for all participants was 70.6 ± 9.4 years. The percentage of subjects with MetS was 12.1% (14.0% in male, 10.6% in female). 

Table 2 shows the BMI- and age-stratified, and unadjusted and multivariable-adjusted ORs and 95% CIs for MetS and each component factors, according to CAOD score categories. The prevalence of MetS was higher in the highest-CAOD-score group than in the lowest-CAOD-score group, and the multivariate-adjusted OR was 1.92 (95% CI 1.17–3.17) in the total individuals. This association was more evident in the older individuals (OR 1.90, 95% CI 1.04–3.46). As a result of stratification by overweight status, the prevalence of dyslipidemia was higher in the highest-CAOD-score group than in the lowest-CAOD-score group in the overweight individuals, and the multivariate-adjusted OR was 2.08 (95% CI 1.17–3.68) (*p* for interaction = 0.02). 

The occurrence of high blood pressure was higher in the highest-CAOD-score group than in the lowest-CAOD-score group in the younger individuals; the multivariate-adjusted OR was 2.02 (95% CI 1.05–3.89). The occurrence of high blood pressure was also higher in the classification that achieved middle-CAOD-score group than in the lowest-CAOD-score group in the younger individuals as evidenced by a multivariate-adjusted OR of 2.28 (95% CI 1.13–4.60).

The prevalence of glucose intolerance was lower in the highest-CAOD-score group than in the lowest-CAOD-score group in the younger individuals; the multivariate-adjusted OR was 0.39 (95% CI 0.17–0.92). 

Table 3 shows unadjusted and multivariable-adjusted ORs and 95% CIs for MetS and each component factors according to CAOD score categories stratified by a combination of overweight status and age categories. The prevalence of dyslipidemia was higher in the highest-CAOD-score group than in the lowest-CAOD-score group in the older and overweight individuals; the multivariate-adjusted OR was 2.11 (95% CI 1.06–4.19). In younger and overweight individuals, the prevalence of dyslipidemia was found to be higher in the middle-CAOD-score group than in the lowest-CAOD-score group. The multivariate-adjusted OR was 4.31 (95% CI 1.13–16.5). 

The prevalence of high blood pressure was also higher in the middle-CAOD-score group than in the lowest-CAOD-score group in only the younger and non-overweight individuals, although the multivariate-adjusted OR was 2.49 (95% CI 1.06–5.84).

## 4. Discussion

In the present study, we demonstrated that the prevalence of MetS in the total individuals and older individuals, dyslipidemia in the overweight individuals, and high blood pressure in the younger individuals were higher in the highest-CAOD-score group than in the lowest-CAOD-score group.

The association between occupational dysfunction and MetS and its component factors has been investigated previously in a workplace setting [18]. However, there has been no study investigating the association between occupational dysfunction and MetS and its component factors among community-dwelling Japanese men and women. This study was the first to examine the association between occupational dysfunction and MetS and its component factors in community-dwelling adults.

An earlier study investigated the relationship between occupational dysfunction and lifestyle behaviors in 65 subjects aged 22–52 years working as rehabilitation therapists in hospitals, where occupational dysfunction was found to be associated with obesity and higher blood pressure [18]. In that study, the correlation coefficient between occupational dysfunction and blood pressure was 0.4 [18]. In the present study, the correlation coefficient between occupational dysfunction and blood pressure was −0.09, and the relationship was weak (*p* = 0.001). The reason for this discrepancy is that the subjects of the previous study were young people with an average age of 27.7 years, who were working and had many social roles. Meanwhile, the subjects of this study were older people with an average age of 70.6 years, who were less likely to be in a full-time job, and had fewer social roles. The mean CAOD score was lower for the older subjects than for the rehabilitation therapists, and the level of occupational dysfunction was mild. We suggest that differences in the characteristics of the subjects caused the difference in results. The lack of habitual exercise and sleep deficiency were associated with occupational dysfunction [18]. People suffering from occupational dysfunction are unable to participate in routine activities of work, leisure, self-care, and rest [23]. The results of this study supported previous research on this score.

In this study, the logistic regression analysis proved that the prevalence of glucose intolerance was lower in the highest-CAOD-score group than in the lowest-CAOD-score group, which is in contrast to our hypothesis. Although there is no clear explanation for this opposite result, it is considered that low reliability accompanying with the small number of subjects may be contributed. Therefore, further investigation is required with a larger number of subjects.

The mechanisms underlying the relationship between occupational dysfunction and MetS, as well as its component factors, are not yet clear. However, generally, it is considered that occupational dysfunction occurs in the early stages of emotional stress [23]. Emotional stress increases blood pressure [26], and makes it easier for a person to eat too much, which in turn results in obesity. Overweight people are more prone to dyslipidemia. Although blood pressure and dyslipidemia are important criteria for the diagnosis of MetS, the authors of this paper believe that occupational dysfunction might also be closely related with MetS. Psychological problems such as depression and stress have been reported to be associated with MetS and cardiovascular disease [27,28,29,30,31]. Therefore, occupational dysfunction may cause the onset of MetS through psychological problems such as depression, and the lack of a sense of purpose in life. The present investigation evidenced that high blood pressure was associated with occupational dysfunction in younger and non-overweight individuals who participated in this study. The fact that younger people are busy with both work and household chores, and that they work long hours every day might contribute to this rise. On the other hand, the problem of high blood pressure may also have amplified in the younger and non-obese population as a result of the increasing Westernization of food habits and the rise in salt intakes in recent years.

Evaluation of occupational dysfunction can be performed conveniently and can be assessed before the development of depression, or burnout syndrome. Therefore, it is considered possible to prevent severe presentations of these diseases. Based on these principles, we actively conduct evaluations and provide guidance on occupational dysfunction during disease prevention and health promotion activities, so that interventions for prevention of disease can begin earlier.

The distinguishing characteristic of this study is that it is the first epidemiological examination of the association between occupational dysfunction and MetS in large community-based population. It can be expected that evaluation of occupational dysfunction will lead to early detection of MetS. Occupational dysfunction may be a new health indicator in the field of preventive medicine. Also, considering the limited number of studies conducted in the Japanese individuals, our study is a great addition to the previous literature.

Several limitations of the study should be considered. First, this is a cross-sectional study and cannot prove causality. It has been reported that falling into occupational dysfunction can cause various health problems including MetS [18,23]. On the other hand, deterioration of a health condition can contribute to occupational dysfunction [16]. Therefore, it is necessary to study the relationship between occupational dysfunction and MetS in detail in longitudinal studies and intervention studies. Second, information on psychological problems such as depression or stress was not obtained in the original health survey. Occupational dysfunction is thought to affect physical diseases through depression and stress, so consideration of these factors is necessary in future analyses. Third, because this research is a survey targeting only one rural area, its generalizability may be limited. Fourth, the subjects of this study were slight-to-moderate individuals of occupational dysfunction compared with previous studies. Finally, although abdominal obesity is vital to the diagnosis of MetS, the authors could not obtain information on waist circumferences for this study. Future investigations should acquire data on waist circumferences.

## 5. Conclusions

Occupational dysfunction was associated with MetS in the total individuals and older individuals, with dyslipidemia in the overweight individuals, and with high blood pressure in the younger individuals. Evaluation and intervention from the viewpoint of occupational dysfunction may be effective in the prevention of MetS. Further research involving longitudinal studies is needed to investigate causality in detail.

## Figures and Tables

**Table 1:** Mean age, age- and sex-adjusted mean values, prevalence of clinical parameters, and lifestyles of subjects.

Variables	All (*n* = 1514)						
	CAOD Score					*p*	*p* for Trend
	16–19	20–30		31≥			
CAOD median	16	24		39			-
Number	489	516		509			-
Male	229	209		220			-
Female	260	307		289			-
Age (years)	72.0	71.3		68.5			<0.0001
BMI (kg/m^2^)	23.0	22.7		22.4	*	**0.007**	0.002
SBP (mmHg)	133.3	131.9		130.5	*	0.069	0.02
DBP (mmHg)	72.8	71.4	*	70.0	**	**<0.0001**	<0.0001
TG (mg/dL)	114.8	114.1		111.1		0.67	0.40
HDL-C (mg/dL)	56.4	57.3		57.7		0.303	0.13
LDL-C (mg/dL)	126.6	122.2	*	121.4	**	**0.011**	0.005
HbA1c (%)	5.7	5.7		5.7		0.411	0.81
Metabolic syndrome, %	12.5	9.7		14.2		0.175	0.67
Current Smoker, %	7.0	5.6		10.0		0.226	0.22
Regular Alcohol intake, %	20.7	20.5		21.0		0.643	0.67
Exercise habits, %	54.6	53.3	**	44.8	**	**0.003**	0.002
Deficiency of sleep, %	15.1	28.9	**	38.9	**	**0.003**	<0.0001
High school or higher education, %	74.9	76.4		77.4		0.893	0.89
Marital status (married), %	75.7	76.6		75.4		0.807	0.97

CAOD: Classification and Assessment of Occupational Dysfunction; BMI: Body Mass Index; SBP: Systolic blood pressure; DBP: Diastolic blood pressure; TG: triglyceride; HDL-C: high-density lipoprotein cholesterol; LDL-C: low-density lipoprotein cholesterol; Metabolic syndrome: BMI ≥ 25 and two or more of following factors: (1) HDL-C of <40 mg/dl and/or TG of ≥150 mg/dl and/or medication for dyslipidemia; (2) SBP ≥ 130 mmHg and/or DBP ≥ 85 mmHg and/or medication for hypertension; and (3) HbA1c ≥ 6.0; prevalence of clinical parameters and life styles of all subjects adjusted by age and sex. Significant differences (*p* < 0.05) by age and sex-adjusted ANCOVA and linear trend among the values of several clinical parameters in CAOD score categories. After ANCOVA, multiple comparisons (Bonferroni) were performed. ** *p* < 0.01, * *p* < 0.05 through multiple comparisons.

**Table 2:** Multivariable-adjusted odds ratios and 95% confidence intervals for metabolic syndrome and each of its component factors according to CAOD score categories in all participants and stratified by overweight status and age categories.

			Total (n = 1514)			
			CAOD Score			*p* for Trend
			16–19	20–30	≥31	
Number			489	516	509	
**Total**						
	Metabolic syndrome					
		No.	61	50	72	
		unadjusted OR (95% CI)	1.00	0.75 (0.51–1.12)	1.16 (0.80–1.67)	0.24
		Multivariable OR (95% CI)	1.00	0.79 (0.47–1.33)	**1.92 (1.17–3.17)**	**0.003**
	High blood pressure					
		No.	319	349	319	
		unadjusted OR (95% CI)	1.00	1.11 (0.86–1.45)	0.90 (0.69–1.16)	0.27
		Multivariable OR (95% CI)	1.00	1.20 (0.91–1.58)	1.12 (0.84–1.48)	0.58
	Dyslipidemia					
		No.	201	208	203	
		unadjusted OR (95% CI)	1.00	0.97 (0.75–1.25)	0.95 (0.74–1.22)	0.71
		Multivariable OR (95% CI)	1.00	1.01 (0.78–1.30)	0.99 (0.76–1.30)	0.92
	Glucose intolerance					
		No.	120	97	107	
		unadjusted OR (95% CI)	1.00	**0.71 (0.53–0.96)**	0.82 (0.61–1.10)	0.32
		Multivariable OR (95% CI)	1.00	0.75 (0.55–1.03)	0.98 (0.71–1.34)	0.88
**BMI < 25 (n = 1179)**						
	Metabolic syndrome					
		No.	–	–	–	
		unadjusted OR (95% CI)	–	–	–	
		Multivariable OR (95% CI)	–	–	–	
	High blood pressure					
		No.	237	270	238	
		unadjusted OR (95% CI)	1.00	1.15 (0.86–1.54)	0.86 (0.64–1.14)	0.17
		Multivariable OR (95% CI)	1.00	1.25 (0.92–1.70)	1.08 (0.79–1.48)	0.96
	Dyslipidemia					
		No.	147	154	136	
		unadjusted OR (95% CI)	1.00	0.94 (0.71–1.26)	0.80 (0.60–1.07)	0.12
		Multivariable OR (95% CI)	1.00	0.94 (0.70–1.27)	0.79 (0.57–1.07)	0.05
	Glucose intolerance					
		No.	78	68	65	
		unadjusted OR (95% CI)	1.00	0.76 (0.53–1.10)	0.74 (0.51–1.06)	0.14
		Multivariable OR (95% CI)	1.00	0.82 (0.57–1.19)	0.90 (0.61–1.32)	0.51
**BMI ≥ 25 (n = 335)**						
	Metabolic syndrome					
		No.	61	50	72	
		unadjusted OR (95% CI)	1.00	0.74 (0.44–1.25)	1.68 (0.98–2.87)	0.03
		Multivariable OR (95% CI)	1.00	0.85 (0.49–1.47)	2.07 (1.15–3.71)	0.01
	High blood pressure					
		No.	82	79	81	
		unadjusted OR (95% CI)	1.00	1.03 (0.58–1.83)	1.12 (0.63–2.02)	0.69
		Multivariable OR (95% CI)	1.00	1.04 (0.57–1.91)	1.27 (0.68–2.38)	0.53
	Dyslipidemia					
		No.	54	54	67	
		unadjusted OR (95% CI)	1.00	1.09 (0.65–1.84)	**1.76 (1.04–2.99)**	**0.03**
		Multivariable OR (95% CI)	1.00	1.28 (0.74–2.21)	**2.08 (1.17–3.68)**	**0.01**
	Glucose intolerance					
		No.	42	29	42	
		unadjusted OR (95% CI)	1.00	0.62 (0.35–1.10)	1.07 (0.63–1.84)	0.56
		Multivariable OR (95% CI)	1.00	0.61 (0.34–1.10)	1.13 (0.64–1.99)	0.47
**Age < 65 (n = 301)**						
	Metabolic syndrome					
		No.	9	11	24	
		unadjusted OR (95% CI)	1.00	0.98 (0.38–2.53)	1.40 (0.61–3.20)	0.32
		Multivariable OR (95% CI)	1.00	1.34 (0.40–4.44)	2.34 (0.78–7.08)	0.36
	High blood pressure					
		No.	33	52	72	
		unadjusted OR (95% CI)	1.00	1.66 (0.89–3.13)	1.18 (0.67–2.10)	0.99
		Multivariable OR (95% CI)	1.00	**2.28 (1.13–4.60)**	**2.02 (1.05–3.89)**	**0.34**
	Dyslipidemia					
		No.	29	35	50	
		unadjusted OR (95% CI)	1.00	0.96 (0.51–1.81)	0.79 (0.44–1.41)	0.37
		Multivariable OR (95% CI)	1.00	1.10 (0.55–2.18)	0.76 (0.40–1.47)	0.17
	Glucose intolerance					
		No.	18	11	14	
		unadjusted OR (95% CI)	1.00	**0.42 (0.18–0.96)**	**0.32 (0.15–0.69)**	**0.01**
		Multivariable OR (95% CI)	1.00	0.51 (0.21–1.25)	**0.39 (0.17–0.92)**	**0.02**
**Age ≥ 65 (n = 1213)**						
	Metabolic syndrome					
		No.	52	39	48	
		unadjusted OR (95% CI)	1.00	0.71 (0.46–1.10)	1.06 (0.70–1.61)	0.59
		Multivariable OR (95% CI)	1.00	0.78 (0.43–1.42)	**1.90 (1.04–3.46)**	0.68
	High blood pressure					
		No.	286	297	247	
		unadjusted OR (95% CI)	1.00	1.05 (0.78–1.40)	0.95 (0.70–1.28)	0.68
		Multivariable OR (95% CI)	1.00	1.08 (0.80–1.45)	1.00 (0.73–1.37)	0.68
	Dyslipidemia					
		No.	172	173	153	
		unadjusted OR (95% CI)	1.00	0.97 (0.74–1.28)	1.02 (0.77–1.36)	0.84
		Multivariable OR (95% CI)	1.00	1.00 (0.75–1.32)	1.07 (0.79–1.45)	0.97
	Glucose intolerance					
		No.	102	86	93	
		unadjusted OR (95% CI)	1.00	0.78 (0.56–1.08)	1.05 (0.76–1.45)	0.57
		Multivariable OR (95% CI)	1.00	0.81 (0.58–1.13)	1.17 (0.83–1.65)	0.46

Multivariable OR: adjusted for sex, age, BMI, smoking habits, regular alcohol intake, sleep, exercise habits, marital status, and education status; data are reported as odds ratio (95% confidence interval); high blood pressure refers to systolic blood pressure ≥ 130 mmHg, diastolic blood pressure ≥ 85 mmHg, and/or medication for hypertension; dyslipidemia denotes high-density lipoprotein cholesterol < 40 mg/dL, triglycerides ≥150 mg/dL, and/or medication for dyslipidemia; glucose intolerance is signified by HbA1c ≥ 6.0% (NGSP) and/or medication for glucose intolerance; CAOD: Classification and Assessment of Occupational Dysfunction; OR: odds ratio; CI: confidence interval; BMI: body mass index.

**Table 3:** Multivariable-adjusted odds ratios and 95% confidence intervals for metabolic syndrome and each of its component factors according to CAOD score categories stratified by a combination of overweight status and age categories.

			Total (*n* = 1514)			
			CAOD Score			*p* for Trend
			16–19	20–30	≥31	
Number			489	516	509	
**Age ≥ 65 years and BMI < 25 (*n* = 972)**						
	Metabolic syndrome					
		No.	–	–	–	
		unadjusted OR (95% CI)	–	–	–	
		Multivariable OR (95% CI)	–	–	–	
	High blood pressure					
		No.	219	238	196	
		unadjusted OR (95% CI)	1.00	1.09 (0.79–1.50)	0.94 (0.67–1.31)	0.62
		Multivariable OR (95% CI)	1.00	1.13 (0.81–1.58)	1.00 (0.71–1.42)	0.74
	Dyslipidemia					
		No.	126	133	108	
		unadjusted OR (95% CI)	1.00	1.00 (0.73–1.36)	0.90 (0.65–1.25)	0.51
		Multivariable OR (95% CI)	1.00	0.98 (0.71–1.35)	0.90 (0.64–1.27)	0.37
	Glucose intolerance					
		No.	68	63	63	
		unadjusted OR (95% CI)	1.00	0.85 (0.58–1.24)	1.02 (0.69–1.50)	0.81
		Multivariable OR (95% CI)	1.00	0.89 (0.60–1.32)	1.12 (0.75–1.68)	0.68
**Age ≥ 65 years and BMI ≥ 25 (*n* = 241)**						
	Metabolic syndrome					
		No.	52	39	48	
		unadjusted OR (95% CI)	1.00	0.68 (0.37–1.24)	1.80 (0.92–3.51)	0.06
		Multivariable OR (95% CI)	1.00	0.72 (0.38–1.35)	2.01 (0.99–4.06)	0.04
	High blood pressure					
		No.	67	59	51	
		unadjusted OR (95% CI)	1.00	0.92 (0.47–1.80)	1.08 (0.52–2.21)	0.82
		Multivariable OR (95% CI)	1.00	0.84 (0.41–1.71)	1.07 (0.50–2.29)	0.93
	Dyslipidemia					
		No.	46	40	45	
		unadjusted OR (95% CI)	1.00	0.93 (0.51–1.69)	**1.91 (1.00–3.66)**	**0.04**
		Multivariable OR (95% CI)	1.00	1.04 (0.56–1.95)	**2.11 (1.06–4.19)**	**0.03**
	Glucose intolerance					
		No.	34	23	30	
		unadjusted OR (95% CI)	1.00	0.65 (0.34–1.24)	1.32 (0.70–2.51)	0.30
		Multivariable OR (95% CI)	1.00	0.62 (0.32–1.21)	1.26 (0.64–2.47)	0.39
**Age < 65 years and BMI < 25 (*n* = 207)**						
	Metabolic syndrome					
		No.	–	–	–	
		unadjusted OR (95% CI)	–	–	–	
		Multivariable OR (95% CI)	–	–	–	
	High blood pressure					
		No.	18	32	42	
		unadjusted OR (95% CI)	1.00	1.84 (0.85–4.00)	1.17 (0.57–2.37)	0.84
		Multivariable OR (95% CI)	1.00	**2.49 (1.06–5.84)**	1.87 (0.83–4.22)	0.50
	Dyslipidemia					
		No.	21	21	28	
		unadjusted OR (95% CI)	1.00	0.67 (0.31–1.46)	0.48 (0.23–0.99)	**0.05**
		Multivariable OR (95% CI)	1.00	0.67 (0.28–1.58)	0.35 (0.15–0.82)	**0.007**
	Glucose intolerance					
		No.	–	–	–	
		unadjusted OR (95% CI)	–	–	–	
		Multivariable OR (95% CI)	–	–	–	
**Age < 65 years and BMI ≥ 25 (*n* = 94)**						
	Metabolic syndrome					
		No.	9	11	24	
		unadjusted OR (95% CI)	1.00	1.08 (0.35–3.31)	2.22 (0.80–6.21)	0.08
		Multivariable OR (95% CI)	1.00	2.25 (0.57–8.89)	3.84 (0.91–16.17)	0.07
	High blood pressure					
		No.	15	20	30	
		unadjusted OR (95% CI)	1.00	1.50 (0.47–4.81)	1.50 (0.52–4.35)	0.54
		Multivariable OR (95% CI)	1.00	1.80 (0.48–6.74)	2.94 (0.75–11.52) a	0.15
	Dyslipidemia					
		No.	8	14	22	
		unadjusted OR (95% CI)	1.00	2.00 (0.65–6.17)	2.20 (0.78–6.24)	0.20
		Multivariable OR (95% CI)	1.00	**4.31 (1.13–16.5)**	3.11 (0.84–11.48)	0.17
	Glucose intolerance					
		No.	8	6	12	
		unadjusted OR (95% CI)	1.00	0.55 (0.16–1.88)	0.80 (0.27–2.36)	0.90
		Multivariable OR (95% CI)	1.00	0.70 (0.18–2.74)	0.61 (0.17–2.22)	0.49

Multivariable OR: adjusted for sex, age, BMI, smoking habits, regular alcohol intake, sleep, exercise habits, marital status, and education status; a; adjusted for age, BMI, smoking habits, regular alcohol intake, sleep, exercise habits, marital status; data are reported as odds ratio (95% confidence interval); high blood pressure refers to systolic blood pressure ≥ 130 mmHg, diastolic blood pressure ≥ 85 mmHg and/or medication for hypertension; dyslipidemia denotes high-density lipoprotein cholesterol < 40 mg/dL, triglycerides ≥ 150 mg/dL and/or medication for dyslipidemia; glucose intolerance is taken as HbA1c ≥ 6.0% (NGSP) and/or medication for glucose intolerance. CAOD: Classification and Assessment of Occupational Dysfunction; OR: odds ratio; CI: confidence interval; BMI: body mass index.
